# Difference in Response to a Motor Imagery Task: A Comparison between Individuals with and without Painful Temporomandibular Disorders

**DOI:** 10.1155/2018/6810412

**Published:** 2018-07-30

**Authors:** Daisuke Uritani, Tomoko Nishida, Nanami Sakaguchi, Tetsuji Kawakami, Lester E. Jones, Tadaaki Kirita

**Affiliations:** ^1^Department of Physical Therapy, Faculty of Health Science, Kio Univeristy, 4-2-2 Umaminaka, Koryocho, Kitakatsuragigun, Nara 6350832, Japan; ^2^Department of Rehabilitation, Japanese Red Cross Kyoto Daiichi Hospital, 15-749 Honmachi, Higashiyamaku, Kyoto 6050981, Japan; ^3^Department of Rehabilitation, Higashiosaka Yamaji Hospital, 1-7-5 Inaba, Higashiosaka, Osaka 5780925, Japan; ^4^Department of Oral and Maxillofacial Surgery, Nara Medical University, 840 Shijo-Cho, Kashihara, Nara 6348521, Japan; ^5^Judith Lumley Centre, La Trobe University, Plenty Road, Bundoora, Melbourne, VIC 3086, Australia

## Abstract

The aim of the study was to investigate the difference in response to a motor imagery task between individuals with and without painful temporomandibular disorders (TMDs). The participants were 24 adults with and without TMD (TMD and control group, resp.). A set of photographic images of the profile view of a person's head and neck and a hand and a foot were presented in a random order. The set consisted of six different orientations with rotations of each image at 0, 60, 120, 180, 240, and 300 degrees and included left and right representations. The participants were required to view the image and make a decision as to whether it was a left or a right side presented, that is, mental rotation (MR) task. Data were collected on 48 tasks (including left and right) at each orientation for each body part. Reaction times (RTs) for correct answers and accuracy in making the left or right judgements were recorded. The RT was slower in the TMD group than in the control group. The RT for the profile image was slower than those for the hand and foot images. For images that were 180 degrees, the RT was slower and the accuracy was lower than those for five of the other image orientations. The judgements made about the 180-degree rotated image were more inaccurate compared to images of all other orientations among all types of stimuli.

## 1. Introduction

Temporomandibular disorder (TMD) is a collective term used for explaining a group of conditions in the temporomandibular joint (TMJ) and its related structures [1]. Orofacial pain is the most common symptom of TMD and is usually concentrated in masticatory muscles and/or TMJ [2]. TMDs are also frequently accompanied by limitation of mouth opening and pain-related disability [2]. Among those with acute presentation of TMD, approximately 15% will develop chronic TMD [3].

A recent review of evidence supports the role of physiotherapy in the treatment of TMD including the use therapeutic exercise [4, 5]. However, in clinical practice, some patients with TMD cannot move their TMJ actively because of pain, restricted range of mouth opening, or fear of movement. The resultant immobility can enhance pain and lead to further restriction to range of movement. As a result, a vicious cycle involving pain and immobility can develop and lead to a persistent pain condition [6].

Mental rotation (MR) describes a motor imagery task [7], where rotated images of a body part are presented and the participant is required to make a judgement about whether the image is of a left side or a right side. The assumption is that participants will make their decision by mentally orientating their own body part to match the picture. Although no actual movement takes place, it appears that MR may be a valuable model for exploring the central processes involved in the control of movement.

In support of this, when MR was used with images of hands [8–11] and feet [12], the relevant area of motor cortex was shown to be activated. When images of the side that corresponds to the person's dominant hand are presented, the judgements tend to be made faster [10]. Interestingly it has also been found that when someone has persistent pain, responses to the MR task are impaired [13–16].

As a result of these findings, MR is considered to have potential for early stage musculoskeletal physiotherapy for patients with motion pain or pain-related fear of movement because it can be performed without movement. In fact, MR has already been successfully applied in physiotherapy for patients with musculoskeletal disorders, especially for extremities [17]. In one example, Moseley [15] described the beneficial effect of motor imagery including MR on functional improvement of hands for CRPS patients.

There are few reports about physiotherapy using motor imagery of the orofacial region. von Piekartz et al. [18] performed a left/right facial posture judgement task using MR. They asked people with chronic facial pain to respond to the images by identifying the positioning of facial features, including jaw or tongue position. In the images, these were positioned either towards the left or right side of the face. The order of presentation of the left and right images was randomly presented and images were also rotated. They concluded that people with chronic facial pain were less accurate than controls at a left/right facial posture judgement task [18].

Regarding TMJ, the region in the human precentral gyrus responsible for the execution of voluntary jaw opening movements has been found to be activated during observation of simple jaw opening movements [19]. In addition, functional magnetic resonance imaging studies in humans have revealed somatotopic activation of the premotor cortex during observation of the actions of the mouth, arm/hand, and foot [20]. In people with TMD, alterations in brain activity have been noted in a range of tasks [21–25]. Hence, it has been considered that MR of a particular part, including TMJ, would activate the motor cortex somatotopically, and there might be value in determining the impact of TMD on performance of a MR task.

Applying MR to TMD patients with orofacial pain presents a challenge. Usually MR of the body parts involves the left-right judgement of disoriented body parts in extremities [26]. However, TMJ has different anatomical and kinesiological characteristics compared to joints in extremities. Also, for axial parts of the body, such as trunk and face, it is less clear how to recognize preferred sides compared to the dominant side in extremities such as hand and foot. Where the task involves the referred or dominant side, there may be an influence on the reaction speed and accuracy of left and right judgements. Identifying how these variations may influence the application of MR to TMD pain needs further clarification.

This study aimed at comparing the responses of participants with and without TMD with orofacial pain when undertaking MR tasks for affected (head and neck profile including TMJ) and nonaffected (hand and foot) body parts. We included MR tasks for hands and feet to discriminate the difference of reaction in MR tasks between affected and nonaffected body parts. Research has demonstrated observation of mouth and hand action activated the mouth- and hand-area of the primary motor cortex, respectively [25, 27, 28]. It was hypothesized that reaction in the MR task for the head and neck profile images would be delayed and inaccurate selectively in the TMD patients compared with the healthy controls.

## 2. Materials and Methods

The participants were 24 adults (23 women) with TMD including orofacial pain, diagnosed based on the criteria for TMD by the Japanese Society for Temporomandibular Joint (TMD group), and 24 adults (21 women) without TMD (control group). No participants in the TMD group had surgery in the orofacial region. Clinical information on the TMD group is provided in Table 1. The Research Ethics Committee of Kio University (H28-59) and Nara Medical University (497-3) approved this study, and all participants provided written informed consent.

Anthropometric data, pain intensity, and passive range of mouth opening (mouth ROM) were collected from medical records. Pain intensity was assessed using a visual analogue scale (VAS), and participants were verbally instructed that the left hand end of the scale represented “no pain” and the right end of the scale represented “worst imaginable pain.” Mouth opening was measured using a standardized measure (Keisei Kaikoudo scale, Keisei Medical Industrial Co., Ltd. Tokyo, Japan) by a dentist.

All participants were tested in the left and right judgements of body parts (head and neck side profiles, hands, and feet) using MR. The order of presentation of these three body parts was determined by a simple process, where the participant blindly selected from a collection of sticks, and each stick was named with a body part.

**Figure 1:**
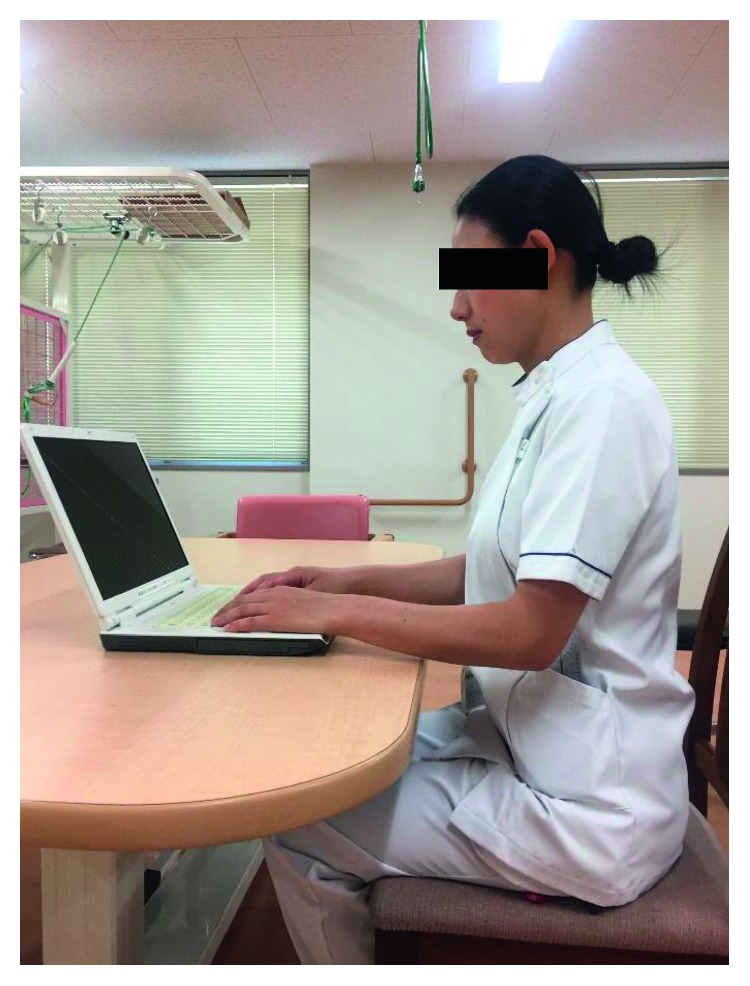
Experimental setting. Participants sat in front of a laptop computer and were asked to put their right and left index fingers on the “M” and “Z” keys, respectively. They were asked to maintain their position, gaze at the screen, and maintain their posture during testing.

The testing was performed in a consultation room in a university hospital. Participants sat in front of a laptop computer (LAVIE HZ750, NEC, Japan, with screen size 16.5 cm by 29.0 cm) and were asked to put their right and left index finger on the “M” and “Z” key, respectively. They were asked to maintain their position, gaze at the screen, and maintain their posture during testing (Figure 1). Participants' viewing distance from the laptop screen and the horizontal screen angle were not set in advance and adjusted so each participant could operate the laptop keyboard comfortably.

**Figure 2:**
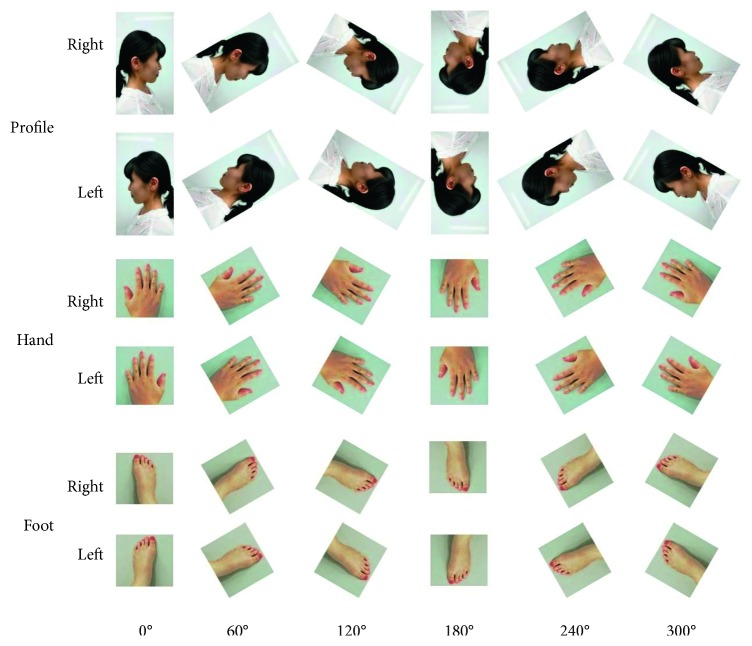
Photographic images of three types of experimental stimuli (profile, hand, and foot) in the six orientations (0°, 60°, 120°, 180°, 240°, and 300°) used in mental rotation. Left stimuli were mirror images of right stimuli.

SuperLab (Cedrus Corporation, USA), psychological experiment software for presenting experimental imagery, was used for the MR task. The images consisted of realistic photos (11 cm square) of a head and neck profile of a young woman, a hand, and a foot presented on the laptop screen (Figure 2). Left and right photographic images were mirror images of each other. Photographic images of hands and feet were taken from the dorsal surface. All stimuli were presented at six different degrees of orientations (0°, 60°, 120°, 180°, 240°, and 300°) for both sides. Photographic images were rotated in a clockwise direction from 0°.

A total of 36 different images, including different orientations, were delivered: 12 for the profile, 12 for the hand, and 12 for the foot image. Testing was completed in three sets so that each body part was tested separately. The sequence of orientation and side of photographic images for each part was randomized between subjects. After each image was presented on the laptop screen, participants had to make a judgement to determine if the image represented a left or right side (i.e., for profile) or a left or right body part (i.e., for hand and for foot). Participants were asked to answer as quickly and accurately as possible using the laptop keyboard. If the answer was right, the participants pressed the “M” key, if left, the participants pressed the “Z” key. Reaction time (RT) was defined as the time between the appearance of the image on the laptop screen and pushing the key. Whether the participant's response was correct or incorrect was also recorded. Stimuli remained on the screen until participants pushed the key. The trigger for computer to present the next picture was when the M or Z key was pressed. As with previous research, if a RT was more than three standard deviations above the mean for a particular stimulus type and image orientation (e.g., profile and 60 degrees), the trial was eliminated and considered as an “incorrect” answer (13 out of 852 trials (1.5%) in the TMD group and 15 out of 864 trials (1.7%) in the control group) [10]. This is because the person has more time to think about the process and so their response to MR is not capturing the desired automatic response. This meant these slow reactions times were not included in the RT analysis, as only correct answers were considered in the RT analysis.

Forty-eight responses, provided by 24 participants for left and right sides, were attained for each cell (defined by stimulus type and side) for the profile and foot stimulus types in both groups and the hand stimulus type in the control group. Only 46 responses, provided by 23 participants, were attained for each cell in the hand stimulus type in the TMD group because the data from one participant in TMD group could not be recorded correctly and were excluded before statistical analysis. Subsequently, accuracy was calculated as the percentage of the number of the correct answers to 48 responses in the profile and foot stimulus type in both groups and the hand stimuli type in the control group and to 46 responses in the hand stimuli in the TMD group. We analyzed RTs and accuracy using analysis of variance (ANOVA) with a post hoc test. When analyzing RTs, ANOVA included a between-subjects factor, “group” (TMD and control), and two within-subjects factors, “stimulus type” (profiles, hands, and feet), and “stimulus orientation” (0°, 60°, 120°, 180°, 240°, and 300°). Post hoc comparisons were carried out using Tukey's test, the Games-Howell test, or unpaired *t* test, when necessary. In addition, we explored correlations between mean RT and accuracy of response for the profile stimulus type, and pain intensity and mouth ROM were calculated in the TMD group. Statistical analysis was performed using IBM SPSS statistics 22 (IBM Japan, Tokyo, Japan). Significance level was set at 5%.

## 3. Results

Participants' characteristics for each group are presented in Table 1. The participants were mostly women with a right sided dominance. The TMD group mean pain for the VAS was 39.4 (standard deviation 30.2). Predictably, mouth ROM in the TMD group was smaller than that in the control group (*p* < 0.05).

### 3.1. Reaction Time to Mental Rotation (MR) Task

Left and right judgement RTs of the two groups are presented in Table 2. The following highlights the significant results drawing on information from both of these tables.

In ANOVA for the group factor, RT of the TMD group (1333.8 ± 702.8 ms) was significantly slower than that of the control group (1161.3 ± 517.6 ms) (*F* (1. 1478) = 43.1, *p* < 0.01). Regarding the stimulus type factor, RT for profile (1371.0 ± 743.6 ms) was significantly slower than that for hand (1252.8 ± 601.2 ms) and foot (1128.9 ± 483.9 ms), and RT for hand was significantly slower than that for foot (*F* (2. 1478) = 29.0, *p* < 0.01). Regarding the stimulus orientation factor, the overall RT for images rotated by 180 degrees (1722.3 ± 818.3 ms) was significantly slower than RTs for five of the other image orientations (0 degree = 1054.3 ± 457.9 ms; 60 degrees = 1104.1 ± 444.3 ms; 120 degrees = 1344.9 ± 713.4 ms; 240 degrees = 1308.6 ± 612.0 ms; 300 degrees = 1101.8 ± 459.9 ms). RTs for 120 and 240 degrees were significantly slower than those for 0, 60, and 300 degrees (*F* (5. 1478) = 45.2, *p* < 0.01).

**Figure 3:**
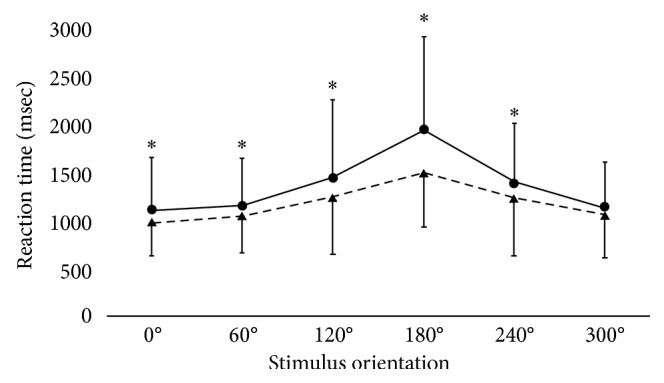
Mean reaction times of three stimulus types of TMD group (circles) and control group (triangle). Error bars indicate standard deviations. Asterisks indicate significant differences between two groups (*p* < 0.05).

The interaction between group and stimulus orientation factors was significant (*F* (5. 1478) = 3.1, *p*=0.01). The TMD group was slower than the control group at all stimulus orientations except 300 degrees (Figure 3). Within both groups, RT for 180 degrees was slower than those for all other image orientations. Within the TMD group, RTs for 120 and 240 degrees were slower than those for 0, 60, and 300 degrees. Within the control group, RTs for 120 and 240 degrees were slower than those for 0 and 60 degrees (Figure 3). Group and stimulus type factors, stimulus type and stimulus orientation factors, and group, stimulus type, and stimulus orientation factors were not significant (*F* (2. 1478) = 0.2, *p*=0.82; *F* (10. 1478) = 1.8, *p*=0.06; *F* (10. 1478) = 0.7, *p*=0.68, resp.).

Mean RT for profile in the TMD group was significantly correlated with mouth ROM (*r*=0.42, *p*=0.04), but not with pain intensity (*r*=−0.24, *p*=0.25).

### 3.2. Accuracy in Response to Mental Rotation (MR) Task

Accuracies are presented in Table 3. For the stimulus type factor, the accuracy for profile (83.2%) and hand (86.9%) was significantly lower than that for foot (94.6%) (*F* (2. 27) = 14.5, *p* < 0.01). In the stimulus orientation factor, the accuracy for 180 degrees (65.0%) was significantly lower than those for all other image orientations (0 degree = 94.4%; 60 degrees = 96.2%; 120 degrees = 90.6%; 240 degrees = 87.0%; 300 degrees = 96.2%) (F (5. 27) = 30.2, *p* < 0.01). No significant difference was presented in the group factor (TMD group = 86.9%; control group = 89.6%) (*F* (1. 27) = 2.4, *p*=0.14).

No significant differences were found between the TMD group (86.9%) and the control group (89.6%) for accuracy. Mean accuracy for profile stimulus type in the TMD group was not correlated with mouth ROM (*r*=−0.06, *p*=0.79) or pain intensity (*r*=−0.20, *p*=0.35).

## 4. Discussion

The result in this study showed that the RT in the MR was slower in the TMD group than in the control group. In addition, the RT for profiles was slower than those for hands and feet. Furthermore, for the inverted image (i.e., 180 degree rotation), the RT was slower, and the accuracy was lower than those for more familiar orientations (i.e., more upright). The response for the inverted image was more inaccurate compared to images of all other orientations, among all stimulus types. The significant interaction between group and stimulus orientation factors means between-group differences occur only at specific degrees of image orientation. In this study, RT between the TMD and the control group presented significant difference at 0 to 240 degrees orientation, but not at 300 degrees orientation. This result could be expected given what is known of brain activation in people with TMD [21–25].

Mouth ROM in the TMD group was smaller than that in the control group. Average heights in both group did not have a significant difference, though average weights did. It is inferred that normal mouth ROM is associated with height rather than weight. Therefore, it is considered that the TMD group had functional limitation in the TMJ compared with the control group because average heights had no significant difference.

Previous studies described the delayed RT and/or decreased accuracy in MR of body parts in people with chronic leg pain [12], chronic shoulder/arm pain [13], and other musculoskeletal pain and dysfunction, such as complex regional pain syndrome [14], carpal tunnel syndrome [16], and cervical dystonia [10]. The results in this study correspond to the results in these studies. Therefore, as has been shown with painful conditions in the extremities, painful TMD appears to disturb reaction in MR compared with healthy controls.

It might be expected that response to MR tasks is affected for the stimulus type associated with the area of pain (e.g., profile and painful TMD). Previous research has shown delayed reaction in MR was found in the affected quadrant selectively among people with pain in extremities [14, 16]. However, in this study, RT in the TMD group was slower, not only for the profile stimulus type but also for hand and foot stimulus types, compared to the control group. Similarly, Fiorio et al. [10] revealed that the response to MR by people with cervical dystonia was affected not only for face but also for hand and foot. Therefore, processing of left and right judgement in MR may be different between extremities, where the effects might be limited to the affected body part and axial parts of the body, such as TMJ.

The results in this study might result from different processing of motor imagery or body perception between TMJ and extremities. Gandevia and Phegan [29] demonstrated that anesthesia of the lips caused distorted perception of the size of thumbs. People with back or neck pain presented altered pain sensitivity and/or tactile spatial acuity in their extremities [30–32]. Distorted sensory processing in the axial part of the body may have influence on the perception of the orientation of the extremities. This might provide an explanation for the results in this study but needs to be clarified with further research, in particular, an investigation into the explicit link between the left and right judgement process and body perception, in people with TMD.

Shibukawa et al. [25] reported that deficit or marked attenuation of the neuromagnetic responses was found during observing jaw opening movement in TMD group compared to healthy subjects, but no significant difference was found during observing palm-opening movements between two groups. That is, they demonstrated that cortical dysfunction associated with jaw-movement observation was specific phenomena in the patients of TMD. Their result does not correspond with our results. This difference may be due to the difference of the way to present image. Our study applied MR using static photographic images while Shibukawa et al. [25] used dynamic video image. Therefore, there may be different visuomotor integration processes between static and dynamic images among TMD patients though we cannot clarify it in the current study.

The difference of accuracy between groups was not significant, while the RT presented between-group difference. A previous study in people with chronic shoulder/arm pain and complex regional pain syndrome also presented results that showed an increased RT but comparable accuracy compared to the control subjects [13]. The process of left and right judgement in MR of the body parts can be described by 3 stages [16, 26]. At first, people make an initial decision on the side of the body pictured [26]. Second, the decision is followed by a mental rotation of the involved body part into the pictured position [26]. At last, people confirm the accuracy of the initial decision via comparing the mentally rotated body part with the actual image [26]. Weissman-Fogel et al. [24] suggested that the slow behavioral responses in people with TMD may be due to functional deficit in recruitment of attention and/or cognition processing areas. Delayed RT may correspond to a disturbance of the initial decision as a correction during the confirmation stage result in a delayed RT while maintaining accuracy [16].

Mean RT of profile stimulus type in the TMD group showed significant positive correlation with passive range of mouth opening. That is, people with larger range of mouth opening had slower RT when presented with the profile stimulus type. The reason why larger range of mouth opening was related to slower RT was not able to be revealed by the results in this study. Indeed, it might be expected the opposite to be the case in patients where mouth opening is painful. The passive range of mouth measurements used might have excluded any guarding due to pain, and the association between active range of mouth opening and RT of profile stimulus type may have presented different result.

The association between mean RT of profile and pain intensity and that between mean accuracy and passive range of mouth opening and pain intensity were not significant. This is not the first study to report RT and pain intensity are not correlated. Two studies using MR found no correlation between RT and accuracy with pain intensity and disability [16, 33]. These results may imply that pain intensity is not associated with disturbance of motor imagery.

This study has several limitations. First, in the TMD group, we did not analyze the association between the painful side and the side presented in the image. There is some evidence in the motor imagery literature that this may be important when evaluating responses [10]. Second, as we mentioned, we have to clarify the possibility of difference of left and right judgement process and/or body perception between people with TMD and with pain in extremities. For example, sensory testing at peripheral sites should be considered in conjunction with MR in future studies. Third, we did not take into account symptom duration because previous research has described no association between RT and accuracy and symptom duration [16, 33]. However, these studies were not investigating painful TMD, and as there seems to be some differences between responses to MR where the location of pain is axial, compared to in the extremities, we would consider that future research might explore duration of symptoms as an influencing factor on RT and accuracy. Fourth, we did not have the resources to capture brain activity as has been done on some studies involving MR, and this would enhance the data supporting findings using RT and accuracy alone. Fifth, most of participants in this study were women. Sex differences were reported in MR [34]. Therefore, the results in this study may be influenced by the rate of participants of women, and we will have to consider the sex difference in future studies. Finally, the number of participants recruited for this study was relatively small. To have greater confidence in the findings, we will need to calculate and recruit the appropriate sample size. More research on motor imagery tasks and specifically involving participants with TMD will help to guide this.

Despite these limitations, we consider the findings of this study to suggest that the use of motor imagery in physiotherapy, for clinical examination and treatment, in TMD patients with chronic pain may be worth investigating. MR has been used for clinical examination for those with chronic pain in extremities, where patients were slower to identify extremities depicted in postures that they find difficult to attain with their own body part [12, 13]. MR also presented a beneficial effect on pain in the extremities when used in physiotherapy treatment [15]. Therefore, our findings which showed that responses to MR by those with pain in the TMJ may be similar to responses by those with pain in the extremities give support for further clinical research into the use of motor imagery for patients with chronic painful TMD.

## 5. Conclusions

The RTs in the MR for profile were more delayed in the individuals with TMD than in those without TMD. When the MR task involved an inverted image, the reaction time was slower and the accuracy was lower than those for other image orientations. Therefore, TMD may influence the motor imagery in the orofacial region. In addition, MR may be useful for assessment and treatment for the TMD patients as well as people with pain in extremities, but further research is needed.

## Figures and Tables

**Table 1:** Characteristics of the participants.

	TMD group	Control group	Overall
Age (years)	49.2 (11.7)	42.5 (19.2)	45.9 (16.1)
Woman (%)	95.8	83.3	89.6
Right-handed (%)	88.0	96.0	91.7
Right-footed (%)	84.0	96.0	89.6
Height (cm)	156.7 (9.6)	161.0 (7.9)	158.8 (8.9)
Weight (kg)	52.2 (9.9)^*∗*^	58.8 (10.6)	55.5 (10.7)
Range of mouth opening (mm)	39.6 (5.8)^*∗*^	47.3 (6.4)	43.4 (7.2)
Pain described by VAS (mm)	39.4 (30.2)	NA	
Subtype of TMD	Myalgia of the masticatory muscle: 11		
	TMJ disc derangement with reduction: 5		
	TMJ disc derangement without reduction: 2		
	Myalgia + disc derangement with reduction: 2		
	Unknown: 4		

^*∗*^ *p* < 0.05 (TMD group versus control group); TMD, temporomandibular disorders; VAS, visual analog scale; TMJ, temporomandibular joint; NA, not available.

**Table 2:** Mean RTs (msec) in the two groups for each stimulus type and orientation.

	0	60	120	180	240	300	Overall
Profile							
Control	1076.4	1125.8	1383.8	1632.1	1484.9	1113.2	1274.6
TMD	1322.6	1329.0	1698.7	1971.0	1567.3	1222.3	1471.8
Hand							
Control	1037.6	1091.4	1189.5	1602.6	1193.7	1102.6	1175.4
TMD	1076.9	1130.1	1347.2	2304.8	1336.8	1189.4	1336.1
Foot							
Control	858.7	936.1	1169.7	1346.8	1049.3	969.3	1047.2
TMD	965.9	1030.0	1347.5	1701.9	1285.0	1026.2	1212.1
Overall	1054.3	1104.1	1344.9	1722.3	1308.6	1101.8	

**Table 3:** Accuracy (%) in the two groups for each stimulus type and orientation.

	0	60	120	180	240	300	Overall
Profile							
Control	91.7	95.8	83.3	58.3	85.4	95.8	85.1
TMD	93.8	91.7	79.2	50.0	79.2	93.8	81.3
Hand							
Control	93.8	95.8	93.8	58.3	91.7	95.8	88.2
TMD	93.5	95.7	93.5	58.7	76.1	95.7	85.5
Foot							
Control	97.9	97.9	97.9	83.3	95.8	100.0	95.5
TMD	95.8	100.0	95.8	81.3	93.8	95.8	93.8
Overall	94.4	96.2	90.6	65.0	87.0	96.2	

## Data Availability

The data used to support the findings of this study are available from the corresponding author upon request.
